# Detailed structure information a highly thermostable Tb-cluster that based on a prodrug ligand of 2,4,5-trifluoro-3-methoxybenzoic acid

**DOI:** 10.1016/j.dib.2018.08.098

**Published:** 2018-08-30

**Authors:** Chenghui Zeng, Kai Zheng, Xue-Zheng Wang, Hao-Ran Li, Zhi-Peng Zhao, Shengliang Zhong

**Affiliations:** a College of Chemistry and Chemical Engineering, Jiangxi Normal University, Nanchang 330022, PR China; b Qingdao Zhengfangyuanxin Public Health Testing Co., Ltd., Qingdao, PR China

## Abstract

In this brief data article, we present the precise structural information, PARD data and thermographic analysis of the Tb-cluster. Detailed structure, luminescence and detecting properties were discussed in our previous study (Zhao et al., 2017) [1]. The data includes the coordination modes of ligand, PXRD patterns of these Ln-MOFs, thermostability, detailed bond lengths and bond angles of the Tb-cluster.

**Specifications Table**

**Table:** 

Subject area	*Chemistry*
More specific subject area	*Single crystal data of lanthanide complexes*
Type of data	*Table, figure*
How data was acquired	*Crystallography open data base and crystallographic tool–Diamond: Crystallographic Information File Code: 1562500.cif*
Data format	*Analyzed*
Experimental factors	*Single crystal X-ray diffraction data was collected on a Bruker SMART 1000 CCD at 296(2) K, with Mo-Ka radiation (0.71073 Å). The structure was refined by full-matrix least-squares methods with SHELXL-97 module. It crystallizes in Triclinic space group P-1 (no. 2).*
Experimental features	*Block colorless single crystal.*
Data source location	*Jiangxi Normal University, Nanchang, China.*
Data accessibility	*The data are with this article.*
Related research article	*Fei Zhao, Xue-Zheng Wang, Song-Tao He, Pei-Zhi Ma, Wei Zhang, Shu-Juan Zhao, Jun Sun, Crystal Structure and as Highly Sensitive Bifunctional Sensor of a Dinuclear Tb-Cluster, Sensors and Actuators B: Chemical, submission.*

**Value of the data**

• *This data would be valuable for synthesizing highly luminescent lanthanide complexes that based on prodrug Ligand*.
• *This data would be valuable for synthesizing pure bulk samples of lanthanide complexes*.
• *This data provide a new way to synthesize thermostable lanthanide complexes*.

## 1 Data

**Fig. 1:**
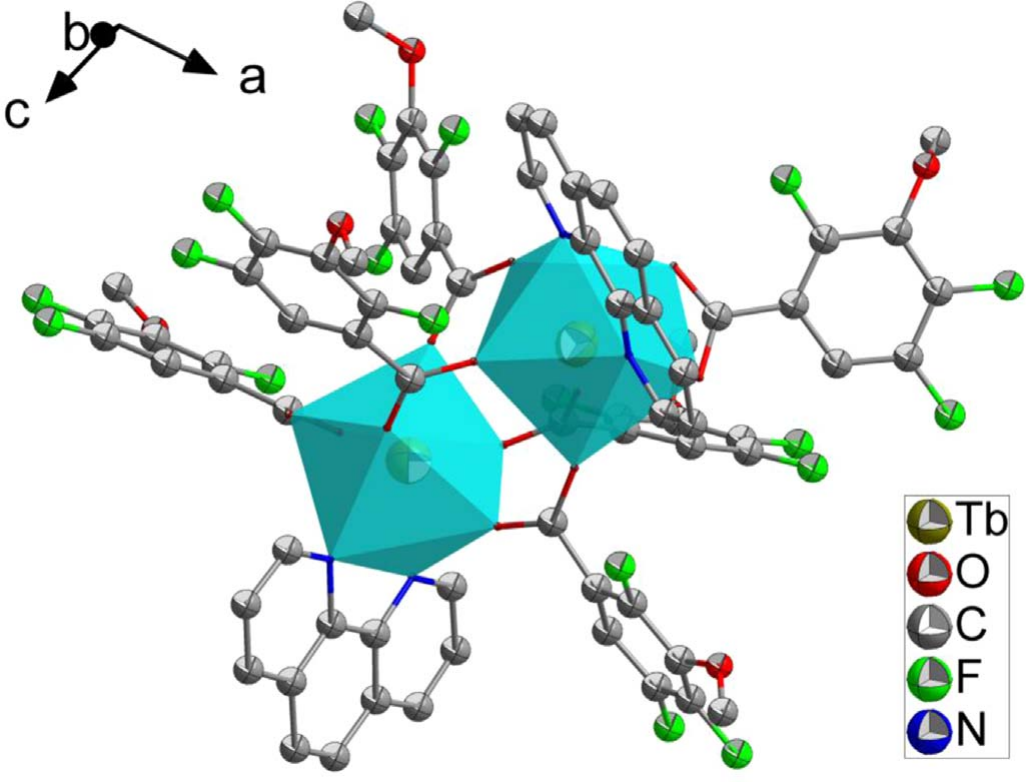
The SBU cluster structure shows the coordination environment of Tb^3+^, two chelated phen arranged at both ends of the dinuclear cluster.

**Fig. 2:**
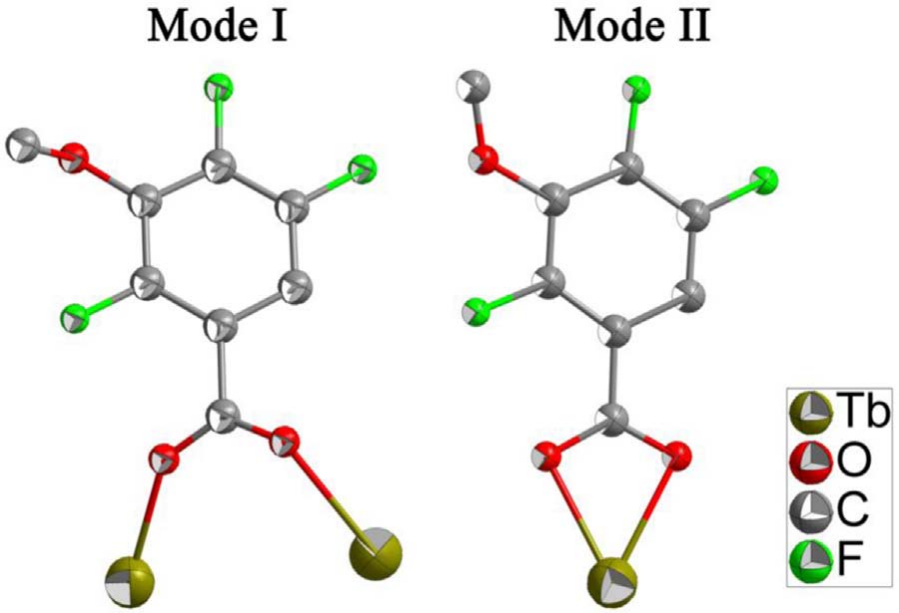
Two coordination modes of prodrug ligand of TFMBA in Tb-cluster.

**Fig. 3:**
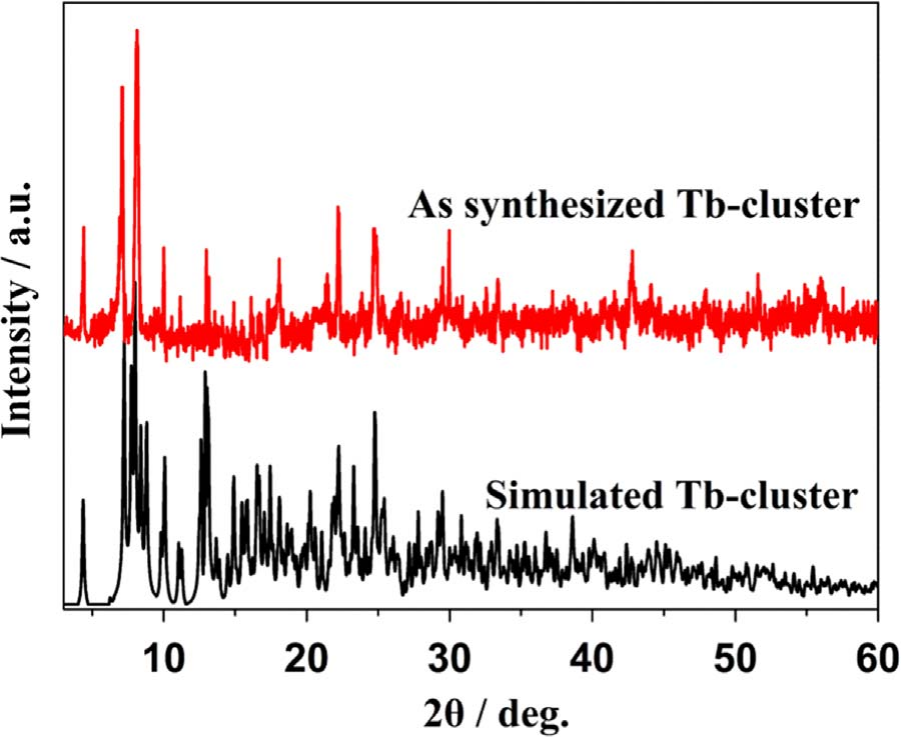
PXRD patterns of as synthesized Tb-cluster competes well with simulated Tb-cluster.

**Fig. 4:**
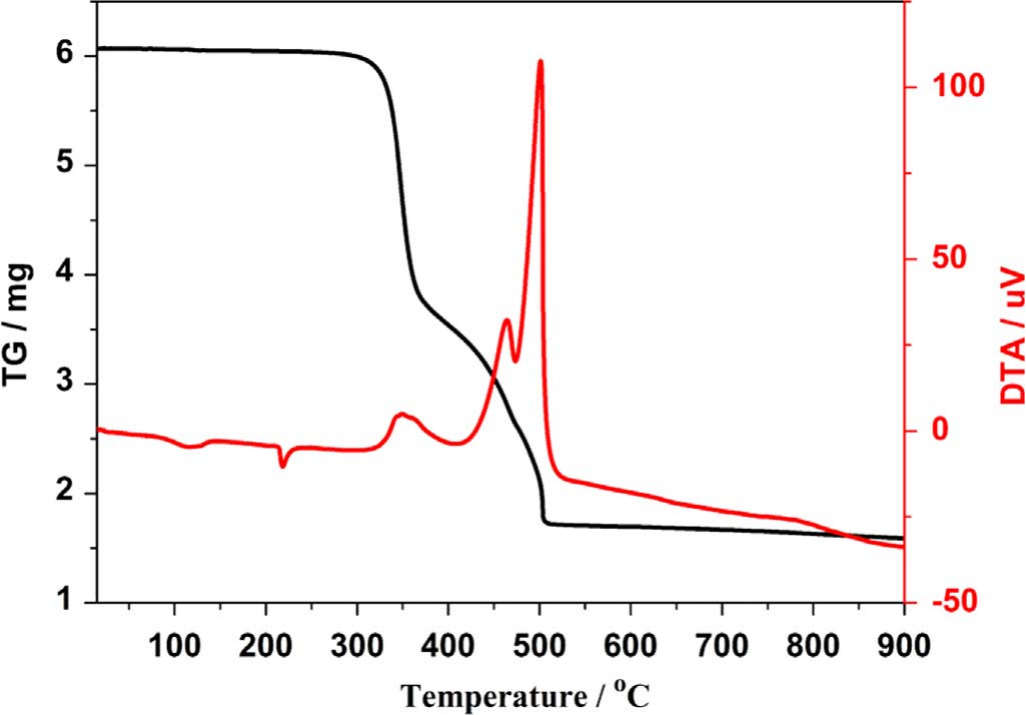
TGA curve of the Tb-cluster which measured in atmosphere.

The crystal structure of Tb-cluster has the chemical formula [1] of [Tb_2_(TFMBA)_6_(phen)_2_] (HTFMBA=2,4,5-Trifluoro-3-methoxybenzoic acid, phen=phenanthroline) [Ln(ADA)_1.5_(phen)]_n_. As shown in Fig. 1, two crystallographically independent Tb^3+^ are bridged by four carboxyls, each dinuclear second building unit (SBU) contains two Tb^3+^, two phen and six fully deprotonated TFMBA, forming a electroneutral unit. Two phen arranged at both ends of the Tb-cluster. The prodrug of TFMBA has two coordination modes of bridge (mode I in Fig. 2) and chelation (mode II in Fig. 2). Detailed information about bond lengths and angles for Tb-cluster are listed in Table 1, it shows that the bond lengths and angles are in the normal value as our previous reports [2], [3], [4], [5], [6], [7], [8], [9]. PXRD (Fig. 3) patterns of as synthesized Tb-cluster competes well with simulated result, indicating the bulk sample is highly pure and the synthesis and separation of the Tb-cluster is successful. TGA result indicates the Tb-cluster is highly stable in air (stable up to 203 **°**C), indicating the Tb-cluster has highly thermostability in the atmosphere (Fig. 4).

**Table 1:** Bond length [Å] and bond angles [deg] for Tb-cluster.

Tb(1)–O(2)	2.300(3)	Tb(2)–O(14)	2.280(3)
Tb(1)–O(4)	2.341(3)	Tb(2)–O(7)	2.296(3)
Tb(1)–O(13)	2.359(3)	Tb(2)–O(5)	2.366(3)
Tb(1)–O(8)	2.398(3)	Tb(2)–O(1)	2.381(3)
Tb(1)–O(16)	2.427(3)	Tb(2)–O(11)	2.423(3)
Tb(1)–O(17)	2.430(3)	Tb(2)–O(10)	2.446(3)
Tb(1)–N(1)	2.553(4)	Tb(2)–N(3)	2.525(3)
Tb(1)–N(2)	2.578(3)	Tb(2)–N(4)	2.609(3)
O(1)–C(1)	1.255(5)	F(16)–C(43)	1.337(6)
O(2)–C(1)	1.253(5)	C(47)–C(42)	1.398(7)
C(1)–C(2)	1.500(6)	C(43)–C(42)	1.389(7)
C(2)–C(3)	1.382(7)	O(18)–C(48)	1.277(9)
C(2)–C(7)	1.382(7)	C(42)–C(41)	1.503(6)
C(3)–F(1)	1.335(6)	O(13)–C(33)	1.252(5)
C(3)–C(4)	1.394(7)	O(14)–C(33)	1.243(5)
C(4)–O(3)	1.357(7)	C(33)–C(34)	1.506(6)
C(4)–C(5)	1.363(8)	C(34)–C(35)	1.374(7)
C(5)–F(2)	1.354(6)	C(34)–C(39)	1.401(7)
C(5)–C(6)	1.373(8)	C(39)–C(38)	1.383(7)
C(7)–C(6)	1.368(7)	C(35)–F(13)	1.347(7)
C(7)–H(7A)	0.9300	C(35)–C(36)	1.404(7)
O(3)–C(8)	1.374(9)	C(36)–O(15)	1.357(8)
F(3)–C(6)	1.354(7)	C(36)–C(37)	1.369(9)
O(4)–C(9)	1.258(5)	O(17)–C(41)	1.256(6)
O(5)–C(9)	1.248(5)	C(41)–O(16)	1.264(6)
C(9)–C(10)	1.504(6)	F(15)–C(38)	1.349(7)
C(10)–C(15)	1.382(8)	F(14)–C(37)	1.351(6)
C(10)–C(11)	1.395(8)	O(15)–C(40)	1.318(11)
C(11)–F(4)	1.325(7)	C(38)–C(37)	1.355(9)
C(11)–C(12)	1.403(8)	O(9)–C(24)	1.380(13)
C(15)–C(14)	1.413(8)	O(11)–C(25)	1.253(5)
C(14)–C(13)	1.329(10)	O(10)–C(25)	1.259(5)
C(14)–F(6)	1.346(8)	C(25)–C(26)	1.510(6)
F(5)–C(13)	1.361(7)	C(26)–C(27)	1.381(7)
C(12)–C(13)	1.338(11)	C(26)–C(31)	1.386(7)
C(12)–O(6)	1.467(11)	C(27)–F(10)	1.342(6)
O(6)–C(16)	1.229(11)	C(27)–C(28)	1.399(7)
N(2)–C(60)	1.333(6)	C(31)–C(30)	1.372(7)
N(2)–C(54)	1.360(6)	C(28)–O(12)	1.357(6)
N(1)–C(49)	1.333(6)	C(28)–C(29)	1.378(8)
N(1)–C(53)	1.353(6)	F(11)–C(29)	1.349(6)
C(49)–C(50)	1.403(6)	F(12)–C(30)	1.349(7)
C(53)–C(52)	1.421(7)	O(12)–C(32)	1.378(9)
C(53)–C(54)	1.444(7)	C(29)–C(30)	1.378(8)
C(54)–C(55)	1.405(7)	F(18)–C(46)	1.349(7)
C(60)–C(59)	1.391(7)	F(17)–C(45)	1.348(6)
C(52)–C(51)	1.398(7)	C(46)–C(47)	1.369(7)
C(52)–C(57)	1.433(7)	C(46)–C(45)	1.378(8)
C(55)–C(58)	1.413(8)	C(45)–C(44)	1.381(9)
C(55)–C(56)	1.434(8)	C(44)–O(18)	1.348(8)
C(58)–C(59)	1.358(8)	C(44)–C(43)	1.404(7)
C(58)–H(58A)	0.9300	C(18)–C(19)	1.365(8)
C(51)–C(50)	1.359(7)	C(18)–C(23)	1.393(8)
C(57)–C(56)	1.347(8)	F(7)–C(19)	1.347(7)
N(4)–C(72)	1.324(6)	C(23)–C(22)	1.388(8)
N(4)—C(66)	1.364(6)	C(19)–C(20)	1.399(8)
N(3)–C(61)	1.331(6)	C(21)–C(22)	1.354(9)
N(3)–C(65)	1.359(5)	C(21)–F(8)	1.345(7)
C(61)–C(62)	1.392(7)	C(21)–C(20)	1.377(10)
C(66)–C(67)	1.408(6)	C(22)–F(9)	1.358(8)
C(66)–C(65)	1.439(6)	C(20)–O(9)	1.348(8)
C(65)–C(64)	1.407(6)	O(8)–C(17)	1.248(6)
C(67)–C(70)	1.405(7)	O(7)–C(17)	1.251(5)
C(67)–C(68)	1.436(7)	C(17)–C(18)	1.506(6)
C(64)–C(63)	1.403(7)	C(72)–C(71)	1.399(7)
C(64)–C(69)	1.432(7)	C(71)–C(70)	1.364(8)
C(62)–C(63)	1.367(7)	C(68)–C(69)	1.351(7)
O(2)–Tb(1)–O(4)	111.63(12)	C(44)–C(45)–C(46)	121.1(5)
O(2)–Tb(1)–O(13)	74.52(11)	O(18)–C(44)–C(45)	126.5(5)
O(4)–Tb(1)–O(13)	81.22(12)	O(18)–C(44)–C(43)	116.6(6)
O(2)–Tb(1)–O(8)	76.25(12)	C(45)–C(44)–C(43)	116.8(5)
O(4)–Tb(1)–O(8)	78.84(12)	C(46)–C(47)–C(42)	119.8(5)
O(13)–Tb(1)–O(8)	135.14(11)	F(16)–C(43)–C(42)	120.9(5)
O(2)–Tb(1)–O(16)	85.16(12)	F(16)–C(43)–C(44)	116.2(5)
O(4)–Tb(1)–O(16)	150.00(11)	C(42)–C(43)–C(44)	122.9(5)
O(13)–Tb(1)–O(16)	128.26(11)	C(48)–O(18)–C(44)	127.3(7)
O(8)–Tb(1)–O(16)	81.56(12)	C(43)–C(42)–C(47)	117.9(5)
O(2)–Tb(1)–O(17)	79.74(13)	C(43)–C(42)–C(41)	124.3(5)
O(4)–Tb(1)–O(17)	150.47(11)	C(47)–C(42)–C(41)	117.8(5)
O(13)–Tb(1)–O(17)	75.69(12)	C(33)–O(13)–Tb(1)	133.1(3)
O(8)–Tb(1)–O(17)	130.67(12)	C(33)–O(14)–Tb(2)	143.8(3)
O(16)–Tb(1)–O(17)	53.83(11)	O(14)–C(33)–O(13)	126.2(4)
O(2)–Tb(1)–N(1)	144.58(12)	O(14)–C(33)–C(34)	116.1(4)
O(4)–Tb(1)–N(1)	83.05(12)	O(13)–C(33)–C(34)	117.7(4)
O(13)–Tb(1)–N(1)	76.36(11)	C(35)–C(34)–C(39)	119.4(4)
O(8)–Tb(1)–N(1)	139.15(11)	C(35)–C(34)–C(33)	122.2(5)
O(16)–Tb(1)–N(1)	97.54(12)	C(39)–C(34)–C(33)	118.1(5)
O(17)–Tb(1)–N(1)	73.87(12)	C(38)–C(39)–C(34)	118.1(6)
O(2)–Tb(1)–N(2)	148.97(12)	F(13)–C(35)–C(34)	120.6(4)
O(4)–Tb(1)–N(2)	75.43(11)	F(13)–C(35)–C(36)	117.3(5)
O(13)–Tb(1)–N(2)	136.05(11)	C(34)–C(35)–C(36)	122.1(6)
O(8)–Tb(1)–N(2)	75.70(11)	O(15)–C(36)–C(37)	119.5(6)
O(16)–Tb(1)–N(2)	77.86(11)	O(15)–C(36)–C(35)	123.8(6)
O(17)–Tb(1)–N(2)	109.39(12)	C(37)–C(36)–C(35)	116.5(6)
N(1)–Tb(1)–N(2)	64.37(11)	C(41)–O(17)–Tb(1)	92.3(3)
O(2)–Tb(1)–C(41)	80.67(13)	O(17)–C(41)–O(16)	121.5(4)
O(4)–Tb(1)–C(41)	167.64(13)	O(17)–C(41)–C(42)	117.3(4)
O(13)–Tb(1)–C(41)	101.77(13)	O(16)–C(41)–C(42)	121.2(4)
O(8)–Tb(1)–C(41)	106.20(13)	O(17)–C(41)–Tb(1)	60.9(2)
O(16)–Tb(1)–C(41)	27.02(13)	O(16)–C(41)–Tb(1)	60.7(2)
O(17)–Tb(1)–C(41)	26.83(13)	C(42)–C(41)–Tb(1)	176.4(4)
N(1)–Tb(1)–C(41)	86.01(13)	C(41)–O(16)–Tb(1)	92.2(3)
N(2)–Tb(1)–C(41)	94.66(13)	C(40)–O(15)–C(36)	120.0(7)
O(14)–Tb(2)–O(7)	113.02(13)	F(15)–C(38)–C(37)	119.2(5)
O(14)–Tb(2)–O(5)	76.63(12)	F(15)–C(38)–C(39)	119.6(6)
O(7)–Tb(2)–O(5)	74.65(12)	C(37)–C(38)–C(39)	121.1(6)
O(14)–Tb(2)–O(1)	85.36(13)	C(38)–C(37)–F(14)	119.2(6)
O(7)–Tb(2)–O(1)	75.94(12)	C(38)–C(37)–C(36)	122.6(5)
O(5)–Tb(2)–O(1)	135.69(11)	F(14)–C(37)–C(36)	118.2(6)
O(14)–Tb(2)–O(11)	148.02(12)	O(12)–C(32)–H(32A)	109.5
O(7)–Tb(2)–O(11)	76.81(12)	O(12)–C(32)–H(32B)	109.5
O(5)–Tb(2)–O(11)	77.14(11)	H(32A)–C(32)–H(32B)	109.5
O(1)–Tb(2)–O(11)	126.47(12)	O(12)–C(32)–H(32C)	109.5
O(14)–Tb(2)–O(10)	150.05(12)	H(32A)–C(32)–H(32C)	109.5
O(7)–Tb(2)–O(10)	89.26(12)	H(32B)–C(32)–H(32C)	109.5
O(5)–Tb(2)–O(10)	130.65(11)	O(3)–C(8)–H(8A)	109.5
O(1)–Tb(2)–O(10)	80.85(11)	O(3)–C(8)–H(8B)	109.5
O(11)–Tb(2)–O(10)	53.66(10)	H(8A)–C(8)–H(8B)	109.5
O(14)–Tb(2)–N(3)	82.58(12)	O(3)–C(8)–H(8C)	109.5
O(7)–Tb(2)–N(3)	143.90(12)	H(8A)–C(8)–H(8C)	109.5
O(5)–Tb(2)–N(3)	78.09(11)	H(8B)–C(8)–H(8C)	109.5
O(1)–Tb(2)–N(3)	139.56(11)	O(18)–C(48)–H(48A)	109.5
O(11)–Tb(2)–N(3)	74.38(12)	O(18)–C(48)–H(48B)	109.5
O(10)–Tb(2)–N(3)	90.87(12)	H(48A)–C(48)–H(48B)	109.5
O(14)–Tb(2)–N(4)	74.85(12)	O(18)–C(48)–H(48C)	109.5
O(7)–Tb(2)–N(4)	149.85(12)	H(48A)–C(48)–H(48C)	109.5
O(5)–Tb(2)–N(4)	134.56(12)	H(48B)–C(48)–H(48C)	109.5
O(1)–Tb(2)–N(4)	75.82(11)	O(15)–C(40)–H(40A)	109.5
O(11)–Tb(2)–N(4)	112.63(11)	O(15)–C(40)–H(40B)	109.5
O(10)–Tb(2)–N(4)	76.03(12)	F(3)–C(6)–C(7)	120.4(5)
N(3)–Tb(2)–N(4)	63.79(11)	F(3)–C(6)–C(5)	118.5(5)
O(14)–Tb(2)–C(25)	164.26(13)	C(7)–C(6)–C(5)	121.1(5)
O(7)–Tb(2)–C(25)	81.85(13)	C(17)–O(8)–Tb(1)	123.1(3)
O(5)–Tb(2)–C(25)	103.80(13)	C(17)–O(7)–Tb(2)	167.6(3)
O(1)–Tb(2)–C(25)	104.00(13)	O(7)–C(17)–O(8)	125.1(4)
O(11)–Tb(2)–C(25)	26.75(12)	O(7)–C(17)–C(18)	115.2(4)
O(10)–Tb(2)–C(25)	26.91(12)	O(8)–C(17)–C(18)	119.7(4)
N(3)–Tb(2)–C(25)	82.15(12)	C(19)–C(18)–C(23)	118.7(5)
N(4)–Tb(2)–C(25)	94.93(12)	C(19)–C(18)–C(17)	122.9(5)
C(1)–O(1)–Tb(2)	120.1(3)	C(23)–C(18)–C(17)	118.3(5)
C(1)–O(2)–Tb(1)	161.8(3)	C(22)–C(23)–C(18)	119.1(6)
O(2)–C(1)–O(1)	124.0(4)	F(7)–C(19)–C(18)	120.5(5)
O(2)–C(1)–C(2)	116.9(4)	F(7)–C(19)–C(20)	116.6(5)
O(1)–C(1)–C(2)	119.1(4)	C(18)–C(19)–C(20)	122.8(6)
C(3)–C(2)–C(7)	118.6(4)	C(22)–C(21)–F(8)	119.4(7)
C(3)–C(2)–C(1)	123.5(4)	C(22)–C(21)–C(20)	121.8(6)
C(7)–C(2)–C(1)	117.9(4)	F(8)–C(21)–C(20)	118.7(7)
F(1)–C(3)–C(2)	120.3(4)	C(21)–C(22)–F(9)	119.9(6)
F(1)–C(3)–C(4)	117.4(5)	C(21)–C(22)–C(23)	120.8(6)
C(2)–C(3)–C(4)	122.4(5)	F(9)–C(22)–C(23)	119.3(6)
O(3)–C(4)–C(5)	119.4(5)	O(9)–C(20)–C(21)	121.4(6)
O(3)–C(4)–C(3)	123.4(5)	O(9)–C(20)–C(19)	121.6(7)
C(5)–C(4)–C(3)	117.1(5)	C(21)–C(20)–C(19)	116.7(6)
F(2)–C(5)–C(4)	119.2(5)	C(20)–O(9)–C(24)	111.5(8)
F(2)–C(5)–C(6)	119.4(6)	C(25)–O(11)–Tb(2)	92.7(3)
C(4)–C(5)–C(6)	121.4(5)	C(25)–O(10)–Tb(2)	91.5(3)
C(6)–C(7)–C(2)	119.4(5)	O(11)–C(25)–O(10)	122.0(4)
C(4)–O(3)–C(8)	118.2(6)	O(11)–C(25)–C(26)	117.3(4)
C(9)–O(4)–Tb(1)	144.0(3)	O(10)–C(25)–C(26)	120.7(4)
C(9)–O(5)–Tb(2)	139.2(3)	O(11)–C(25)–Tb(2)	60.5(2)
O(5)–C(9)–O(4)	126.0(4)	O(10)–C(25)–Tb(2)	61.5(2)
O(5)–C(9)–C(10)	117.0(4)	C(26)–C(25)–Tb(2)	177.8(3)
O(4)–C(9)–C(10)	117.0(4)	C(27)–C(26)–C(31)	118.2(4)
C(15)–C(10)–C(11)	118.7(5)	C(27)–C(26)–C(25)	125.1(4)
C(15)–C(10)–C(9)	119.7(5)	C(31)–C(26)–C(25)	116.6(4)
C(11)–C(10)–C(9)	121.4(5)	F(10)–C(27)–C(26)	121.4(4)
F(4)–C(11)–C(10)	120.8(5)	F(10)–C(27)–C(28)	116.3(4)
F(4)–C(11)–C(12)	117.9(6)	C(26)–C(27)–C(28)	122.3(5)
C(10)–C(11)–C(12)	121.3(6)	C(30)–C(31)–C(26)	120.5(5)
C(10)–C(15)–C(14)	117.9(6)	O(12)–C(28)–C(29)	121.7(5)
C(13)–C(14)–F(6)	120.5(6)	O(12)–C(28)–C(27)	120.8(5)
C(13)–C(14)–C(15)	121.7(6)	C(29)–C(28)–C(27)	117.4(5)
F(6)–C(14)–C(15)	117.8(7)	C(28)–O(12)–C(32)	116.3(5)
C(13)–C(12)–O(6)	125.6(7)	F(11)–C(29)–C(28)	118.9(5)
C(13)–C(12)–C(11)	118.2(7)	F(11)–C(29)–C(30)	119.9(5)
O(6)–C(12)–C(11)	115.5(7)	C(28)–C(29)–C(30)	121.1(5)
C(14)–C(13)–C(12)	122.3(6)	F(12)–C(30)–C(29)	119.3(5)
C(14)–C(13)–F(5)	117.8(7)	F(12)–C(30)–C(31)	120.3(5)
C(12)–C(13)–F(5)	119.9(8)	C(29)–C(30)–C(31)	120.4(5)
C(16)–O(6)–C(12)	127.9(10)	F(18)–C(46)–C(47)	120.7(5)
C(60)–N(2)–C(54)	117.9(4)	F(18)–C(46)–C(45)	117.8(5)
C(60)–N(2)–Tb(1)	123.3(3)	C(47)–C(46)–C(45)	121.5(5)
C(54)–N(2)–Tb(1)	118.8(3)	F(17)–C(45)–C(44)	120.9(6)
C(49)–N(1)–C(53)	118.2(4)	F(17)–C(45)–C(46)	118.0(6)
C(49)–N(1)–Tb(1)	121.9(3)	N(3)–C(65)–C(64)	122.7(4)
C(53)–N(1)–Tb(1)	119.9(3)	N(3)–C(65)–C(66)	117.7(4)
N(1)–C(49)–C(50)	123.1(5)	C(64)–C(65)–C(66)	119.6(4)
N(1)–C(53)–C(52)	121.8(4)	C(66)–C(67)–C(70)	117.6(5)
N(1)–C(53)–C(54)	118.5(4)	C(66)–C(67)–C(68)	119.5(5)
C(52)–C(53)–C(54)	119.7(4)	C(70)–C(67)–C(68)	122.9(4)
N(2)–C(54)–C(55)	122.3(4)	C(63)–C(64)–C(65)	117.6(4)
N(2)–C(54)–C(53)	118.3(4)	C(63)–C(64)–C(69)	122.2(4)
C(55)–C(54)–C(53)	119.3(4)	C(65)–C(64)–C(69)	120.3(5)
N(2)–C(60)–C(59)	123.3(5)	C(63)–C(62)–C(61)	119.4(5)
C(51)–C(52)–C(53)	118.0(5)	C(62)–C(63)–C(64)	119.4(5)
C(72)–N(4)–C(66)	117.5(4)	C(70)–C(71)–C(72)	119.3(5)
C(72)–N(4)–Tb(2)

## 2 Experimental design, materials, and methods

Prodrug of HTFMBA (200 mg, 0.97 mmol) and 22 mL H_2_O were mixed in a 100 mL beaker, 0.1 M NaOH solution was added to adjust the pH = 6.0. Tb(NO_3_)_3_·6H_2_O (203.8 mg, 0.45 mmol) and 0.45 mmol (81.1 mg) phen were dissolved in 30 mL anhydrous ethanol. The upward two solutions were mixed together in a beaker. After two months’ slow evaporation at temperature of about 15–32 °C, a lot of colorless block crystals were obtained by filtration. The crystals were washed with 5 mL CH_3_OH three times and dried in a desiccator for 24 h. Yield: 57% based on Tb^3+^ salt. Anal. Calcd (%): C, 45.30; H, 2.112. Found (%): C, 45.57; H, 2.123.

Single crystal X-ray diffraction data was collected on a Bruker SMART 1000 CCD, with Mo-Ka radiation (Wavelength = 0.71073 Å) at room temperature. The structure was refined by full-matrix least-squares methods with SHELXL-97 module. Phase purity of as synthesized sample was determined by PXRD, using a DMAX2200VPC diffractometer, at 30 kV and 30 mA.

## References

[1] F. Zhao, X. Wang, S. He, P. Ma, W. Zhang, S. Zhao, J. SunHighly luminescent Ln-MOFs based on 1,3-adamantanediacetic acid as bifunctional sensor, Sensors and Actuators B: Chemical, submission.

[2] Zeng C.-H., Luo Z., Yao J. (2017). Porous hydrogen-bonded organic-inorganic frameworks: weak interactions and selective dye filtration. CrystEngComm.

[3] Xu S.-S., Tao P., Zeng C.-H., Wang Y., Gao L.-F., Nie Q.-Q. (2016). Lanthanide-pamoate-frameworks: visible light excitation for NIR luminescence. Inorg. Chim. Acta.

[4] Yang M.-Q., Zhou C.-P., Chen Y., Li J.-J., Zeng C.-H., Zhong S. (2017). Highly sensitive and selective sensing of CH_3_Hg^+^ via oscillation effect in Eu-cluster. Sens. Actuators B: Chem..

[5] Yan Z.-Q., Meng X.-T., Su R.-R., Zeng C.-H., Yang Y.-Y., Zhong S. (2015). Basophilic method for lanthanide MOFs with a drug ligand: crystal structure and luminescence. Inorg. Chim. Acta.

[6] Zeng C.-H., Meng X.-T., Xu S.-S., Han L.-J., Zhong S., Jia M.-Y. (2015). A polymorphic lanthanide complex as selective Co^2+^ sensor and luminescent timer. Sens. Actuators B: Chem..

[7] Zeng C.-H., Wang J.-L., Yang Y.-Y., Chu T.-S., Zhong S.-L., Ng S.W. (2014). Lanthanide CPs: the guest-tunable drastic changes of luminescent quantum yields, and two photon luminescence. J. Mater. Chem. C.

[8] Li S.-S., Ye Z.-N., Xu S.-S., Zhang Y.-J., Tao A.-R., Liu M. (2015). Highly luminescent lanthanide CPs based on dinuclear cluster: crystal structure and sensitive Trp sensor. RSC Adv..

[9] Zhu Y.-M., Zeng C.-H., Chu T.-S., Wang H.-M., Yang Y.-Y., Tong Y.-X. (2013). A novel highly luminescent LnMOF film: a convenient sensor for Hg^2+^ detecting. J. Mater. Chem. A.

