# Supplementary material for: Detailed structure information a highly thermostable Tb-cluster that based on a prodrug ligand of 2,4,5-trifluoro-3-methoxybenzoic acid
Source: Data Brief. 2018 Aug 30;20:1244–51. doi: 10.1016/j.dib.2018.08.098 (PMC6143725; doi:10.1016/j.dib.2018.08.098)
Supplement: Supplementary file 1 — Supporting information [file mmc1.docx]

Conflicts of interest

There are no conflicts to declare.
